# The influence of a modified haptic on preventing toric IOL rotation in high rotation risk eyes

**DOI:** 10.1016/j.aopr.2025.11.003

**Published:** 2025-11-05

**Authors:** Shuyu Liu, Chao Chen, Jitong Zhou, Kaiwen Cheng, Yu Du, Jiaqi Meng, Yi Lu, Wenwen He, Xiangjia Zhu

**Affiliations:** a Eye Institute and Department of Ophthalmology, Eye & ENT Hospital, Fudan University, Shanghai, China; b NHC Key laboratory of Myopia and Related Eye Diseases, Key Laboratory of Myopia and Related Eye Diseases, Chinese Academy of Medical Sciences, Shanghai, China; c Key Laboratory of Myopia, Chinese Academy of Medical Science, Shanghai, China; d Shanghai Key Laboratory of Visual Impairment and Restoration, Shanghai, China

**Keywords:** White-to-white, Toric intraocular lens, Rotational stability, C-loop frosted haptic, Plate-haptic

## Abstract

**Purpose:**

To evaluate the rotational stability and astigmatic correction of a C-loop frosted haptic toric intraocular lens (IOL) compared with a plate-haptic toric IOL with/without capsular tension ring (CTR) implantation in cataractous eyes with high rotation risk due to large white-to-white (WTW) distance.

**Methods:**

This retrospective cohort study included 90 cataractous eyes with WTW > 11.8 mm, which received implantation of either a C-loop frosted haptic toric IOL (Group ZCU), a plate-haptic toric IOL (Group 709), or a plate-haptic toric IOL with CTR (Group 709 + CTR). IOL rotation and residual astigmatism were assessed one month postoperatively.

**Results:**

Group ZCU demonstrated significantly better rotational stability, with a mean rotation of 1.87° ± 1.03°, compared to 4.63° ± 2.12° in Group 709 and 4.13° ± 1.86° in Group 709 + CTR (both *P* < 0.05). Residual astigmatism was also significantly lower in Group ZCU (0.36 ± 0.26 D), compared to Group 709 (0.54 ± 0.43 D) and Group 709 + CTR (0.65 ± 0.27 D; both *P* < 0.05). A positive correlation between WTW and IOL rotation was found in Group 709 (*r* = 0.419, *P* = 0.021) and Group 709 + CTR (*r* = 0.403, *P* = 0.027), but not in Group ZCU (*r* = −0.180, *P* = 0.341). Lens thickness and WTW were identified as independent predictors of rotation in Group 709, whereas only WTW was significant in Group 709 + CTR.

**Conclusions:**

In eyes with high rotation risk due to large WTW (> 11.8 mm), the ZCU IOL exhibits superior rotational stability and astigmatic correction compared to the 709 IOL, regardless of whether CTR is used.

## 1 Introduction

Toric intraocular lenses (IOLs) are an effective solution for correcting astigmatism during cataract surgery. By incorporating cylindrical power, toric IOLs can effectively correct corneal astigmatism, significantly improve uncorrected postoperative visual acuity, reduce dependence on spectacles, and enhance overall visual quality.^1^, ^2^, ^3^ However, the effectiveness of toric IOLs in astigmatic correction largely depends on their rotational stability after implantation.^4^, ^5^, ^6^

The rotational stability of toric IOLs is influenced by factors such as haptic design and ocular anatomical characteristics. Among different haptic designs, plate-haptic IOLs, particularly the AT TORBI 709M IOL (709 IOL, Carl Zeiss Meditec AG, Germany), have demonstrated superior rotational stability in myopic eyes with long axial length (AL).^7^ However, our previous study demonstrated that eyes with large white-to-white (WTW) distances, especially those over 11.8 mm, are associated with an increased risk of postoperative rotation, even when plate-haptic toric IOLs are used.^8^ These eyes can be considered as having high rotation risk. To address this issue, capsular tension rings (CTRs) have been introduced to address rotational instability by maintaining the shape of the capsular bag and reducing capsular contraction forces^9^^,^^10^ Nonetheless, the efficacy of CTRs remains controversial. Recently, TECNIS™ Toric II IOL (ZCU IOL, Johnson & Johnson Vision, Irvine, CA, USA), incorporates frosted haptic surfaces to enhance friction between the IOL and the capsular bag,^11^ which was proved to improve rotational stability with average rotation less than 1°. In our clinical experience, we also found its excellent rotational stability. However, the actual clinical performance of ZCU IOL in high rotation risk eyes with large WTW distances remains to be validated.

Therefore, this study investigates the effects of ZCU IOL compared with 709 IOL with or without CTR in high rotation risk eyes (WTW > 11.8 mm), aiming to provide evidence-based guidance for optimizing surgical strategies and enhancing postoperative visual quality.

## 2 Methods

### 2.1 Ethics statement

This retrospective cohort study was approved by the Institutional Review Board of the Eye & ENT (EENT) Hospital of Fudan University, Shanghai, China (Approval No. 2024015-1). The study was registered at https://www.clinicaltrials.gov (accession number NCT02182921) and was conducted under the tenets of the Declaration of Helsinki. Informed consent was obtained from all patients before cataract surgery for using their clinical data.

### 2.2 Patients

Patients who underwent cataract surgery with monofocal toric IOL implantation between Jan 2023 and Sep 2024 at the EENT Hospital of Fudan University were included in this retrospective study. The inclusion criteria were (1) regular corneal astigmatism from 1.0 to 4.0 D (2) WTW > 11.8 mm. The exclusion criteria were (1) lens dislocation or subluxation found by slitlamp examination; (2) obvious zonular weakness found before surgery; (3) other eye disorders (eg, previous trauma, glaucoma, keratoconus et al.); (4) previous ocular surgery histories that could affect the results. If both eyes meet the eligibility criteria, one eye was randomly selected.

Eyes with severe intraoperative or postoperative complications, such as posterior capsule rupture or failure of continuous circular capsulorhexis (CCC) were excluded from analysis as well as eyes without axis marker on the IOL optical surface exposed after full mydriasis. Then the included eyes were divided into three groups according to the types of toric IOLs and if a CTR was used: (1) Group ZCU; (2) Group 709; (3) Group 709 + CTR.

### 2.3 Preoperative examinations

Routine ophthalmic examinations were performed before surgery, including visual acuity, slitlamp examination, biometry measurements (IOLMaster 700, Carl Zeiss Meditec AG, Germany), corneal topography (Pentacam HR, OCULUS Optikgeräte GMBH, Germany), fundoscopy and B-scan ultrasonography. The power and axis of the toric IOL were calculated using the online Barrett toric calculator (https://calc.apacrs.org/toric_calculator20/Toric Calculator.aspx). The predicted postoperative astigmatism was also obtained from the calculator output, based on the input keratometry and selected IOL model.

### 2.4 Surgical procedure

All surgeries were conducted with the image guided Callisto Eye System (Carl Zeiss Meditec AG, Germany) using a standard procedure. A 2.6 mm clear corneal incision was made, followed by a 5.5 mm CCC and phacomulsification. The toric IOL (ZCU or 709) was then implanted into the capsular bag and was aligned to the planned axis. When a 709 IOL was chosen, a CTR may be used or not before IOL implantation. The incisions were hydrated without suture. At the end of the surgery, the final IOL axis was checked and recorded. Routine postoperative anti-inflammation eye drops were administered to all patients after surgery.

### 2.5 Postoperative examinations

Patients were followed up 1 month postoperatively. Only one eye per patient was included, and all postoperative visual and refractive outcomes were evaluated monocularly. Comprehensive ophthalmic examinations were performed, including the measurement of uncorrected (UCVA) and best corrected visual acuity (BCVA), both recorded in logarithm of the minimum angle of resolution (logMAR). Manifest refraction was performed, and the actual postoperative axis of the toric IOL was recorded.

The pupils of all the included eyes were dilated until the axis marker on the toric IOL was visible. Then the retroillumination image of OPD-Scan III aberrometer (Nidek Co, Ltd, Japan) was taken to record the axis of the toric IOL. The degree of toric IOL rotation was calculated as the difference between the actual postoperative axis and the axis recorded at the end of the surgery.

### 2.6 Statistical analysis

The sample size was calculated with G∗Power 3.1 with the effect size of 0.45 according to the pre-test, a significance level of 5% and a power of 95%. According to the calculation, 25 eyes for each group was required. Finally, 90 eyes were included in the analysis, 30 for each group. For the calculation of predicted postoperative astigmatism, the surgically induced astigmatism (SIA) was uniformly set at 0.50 D at 160°, corresponding to the standardized corneal incision used in all surgeries.

Statistical analyses were performed with SPSS Statistics (Version 20, IBM Corp) All continuous data were presented as mean ± standard deviation (SD). One-way analysis of variation (ANOVA) with post hoc Tukey test was used to compare differences between groups for continuous data and Chi-square test was used to compare categorical data. Relationships between continuous variables were assessed with Pearson's correlation analysis. Multiple liner regression was performed to evaluate the independent influencing factors of toric IOL rotation in each group.

## 3 Results

### 3.1 Patient characteristics

A total of 90 eyes were enrolled and divided into three groups, with 30 eyes in each group. The baseline characteristics of the three groups are summarized in Table 1. No significant differences were observed among the groups in terms of age, gender, eye laterality, AL, anterior chamber depth (ACD), lens thickness (LT), WTW and corneal astigmatism. The mean WTW of the three groups was 12.15 ± 0.22, 12.05 ± 0.26, and 12.11 ± 0.23 mm, respectively, with all values ranging from 11.9 to 12.7 mm.

**Table 1:** Baseline characteristics of the three groups.

	Group ZCU	Group 709	Group 709 + CTR	*P* value
Age (years)	59.43 ± 12.63 (39–83)	62.97 ± 10.64 (37–81)	65.27 ± 9.27 (45–78)	0.121
Gender (male/female)	14/16	13/17	12/18	0.877
Eye laterality (right/left)	20/10	17/13	16/14	0.560
AL (mm)	26.21 ± 2.94 (21.8–32.18)	25.82 ± 2.61 (22.38–32.02)	26.97 ± 2.10 (22.61–30.94)	0.219
ACD (mm)	3.42 ± 0.29 (2.68–3.94)	3.32 ± 0.45 (2.61–4.66)	3.54 ± 0.34 (2.93–4.13)	0.087
LT (mm)	4.26 ± 0.49 (3.17–5.14)	4.25 ± 0.57 (2.94–5.07)	4.16 ± 0.34 (3.47–4.72)	0.697
WTW (mm)	12.15 ± 0.22 (11.9–12.7)	12.05 ± 0.26 (11.9–12.7)	12.11 ± 0.23 (11.9–12.7)	0.289
Corneal astigmatism (D)	2.01 ± 0.61 (1.01–3.31)	1.68 ± 0.46 (1.07–3.1)	1.9 ± 0.72 (1.03–3.38)	0.108

AL = axial length; ACD = anterior chamber depth; LT = lens thickness; WTW = white-to-white.

### 3.2 Postoperative visual outcome and refractive astigmatism

The postoperative UCVA, BCVA, predicted refractive astigmatism, and actual postoperative astigmatism were compared across the three groups, as shown in Table 2. Group ZCU demonstrated significantly better mean UCVA (0.21 ± 0.18 logMAR) than the other two groups (0.24 ± 0.15 logMAR for Group 709 and 0.36 ± 0.24 logMAR for Group 709 + CTR, *P* = 0.008). No significant differences in BCVA were found among the three groups (*P* = 0.194).

**Table 2:** Postoperative visual outcome and refractive astigmatism of the three groups.

	Group ZCU	Group 709	Group 709 + CTR	*P* value
UCVA (logMAR)	0.21 ± 0.18	0.24 ± 0.15	0.36 ± 0.24	0.008
BCVA (logMAR)	0.03 ± 0.06	0.04 ± 0.09	0.06 ± 0.06	0.194
Predicted refractive astigmatism (D)	0.20 ± 0.14	0.21 ± 0.09	0.20 ± 0.07	0.932
Actual refractive astigmatism (D)	0.36 ± 0.26	0.56 ± 0.42	0.62 ± 0.24	< 0.05

BCVA=best corrected visual acuity; UCVA=uncorrected visual acuity; logMAR = logarithm of the minimum angle of resolution.

Regarding the predicted refractive astigmatism target, there were no statistically significant differences among the three groups (Group ZCU: 0.20 ± 0.14 D, Group 709: 0.21 ± 0.09 D, Group 709 + CTR: 0.20 ± 0.07 D; *P* = 0.932), indicating comparable surgical planning and baseline expectations.In terms of actual postoperative residual astigmatism, Group ZCU achieved the lowest value (0.36 ± 0.26 D), which was significantly lower than that of Group 709 (0.56 ± 0.42 D) and Group 709 + CTR (0.62 ± 0.24 D) (*P* < 0.05). No significant difference was observed between Group 709 and Group 709 + CTR (*P* = 0.876).

**Fig. 1:**
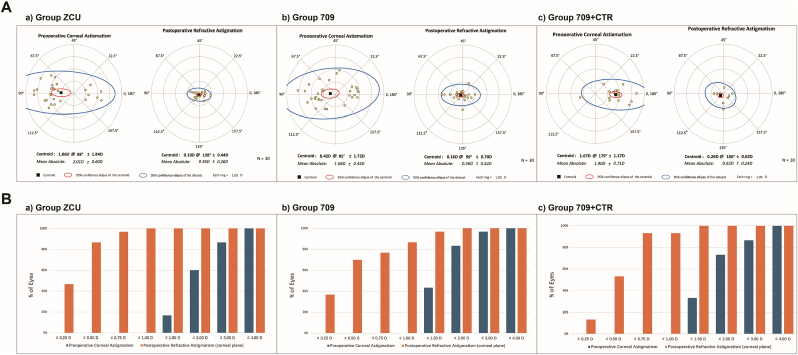
**Comparison of preoperative corneal astigmatism and postoperative refractive astigmatism for the three groups.** A. Double-angle plots showing the 95% confidence intervals (blue ellipse for dataset, red ellipse for centroid) and centroids with standard deviations: Group ZCU: 0.10 at 128° ± 0.44 D, Group 709: 0.16 at 99° ± 0.70 D, Group 709 + CTR: 0.26 at 106° ± 0.62 D. B. Distribution of preoperative and postoperative astigmatism, with blue bars showing preoperative values and orange bars showing postoperative refractive astigmatism. Proportion of eyes with postoperative astigmatism within 0.5 D: 87% in Group ZCU, 70% in Group 709, and 53% in Group 709 + CTR.

**Fig. 2:**
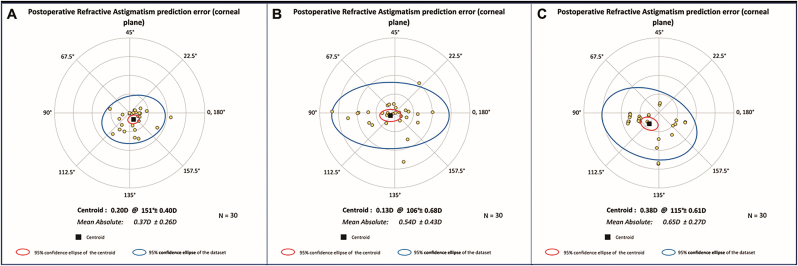
**Double-angle plots showing the refractive astigmatism prediction errors after surgery at the corneal plane across three different groups.** Each dot indicates the refractive outcome of an individual patient, and black squares denote the central tendency (centroid) of the data distribution. The inner red ellipse delineates the 95% confidence area surrounding the centroid, whereas the outer blue ellipse indicates the 95% confidence boundary encompassing the entire data set. Specifically, Group ZCU exhibited a mean absolute error of 0.37 ± 0.26 D (centroid: 0.20 D @151° ± 0.40 D), Group 709 presented a mean absolute error of 0.54 ± 0.43 D (centroid: 0.13 D @106° ± 0.68 D), and Group 709 + CTR demonstrated a mean absolute error of 0.65 ± 0.27 D (centroid: 0.38 D @115° ± 0.61 D).

Fig. 1A shows the double-angle plots of the preoperative corneal astigmatism and postoperative refractive astigmatism at the corneal plane of the three groups. Group ZCU also had the lowest centroid postoperative refractive astigmatism among the three groups (0.10 D @ 128° ± 0.44 D, compared to 0.16 D @ 99° ± 0.70 D and 0.26 D @ 106° ± 0.62 D). Fig. 1B shows the distribution of the preoperative corneal astigmatism and postoperative refractive astigmatism. Group ZCU had more eyes with postoperative refractive astigmatism within 0.5 D than the other two groups (87% compared to 70% and 53%, *P* = 0.019). Additionally, Fig. 2 illustrates that Group ZCU had the lowest mean absolute postoperative refractive astigmatism prediction error among the three groups. The mean absolute error for Group ZCU was 0.37 ± 0.26 D, significantly lower than that of Group 709 (0.54 ± 0.43 D) and Group 709 + CTR (0.65 ± 0.27 D), both *P* < 0.05. The centroid error for Group ZCU was 0.20 D @ 151° ± 0.40 D, compared to 0.13 D @ 106° ± 0.68 D for Group 709 and 0.38 D @ 115° ± 0.61 D for Group 709 + CTR.

### 3.3 Postoperative rotation of toric IOLs

Table 3 presents comparisons of total, clockwise and counterclockwise postoperative rotation among the three groups. The mean rotation of the toric IOL was significantly lower in Group ZCU than in the other two groups (1.87 ± 1.63° vs 4.63 ± 4.03° and 4.13 ± 2.10°, both *P* < 0.05), and no significant difference was found between the other two groups. IOL rotation of 5.0° or less was observed in 29 eyes (96.7%) in Group ZCU, which was significantly higher compared to 23 eyes (76.7%) in Group 709 and 20 eyes (66.7%) in Group 709 + CTR (*P* = 0.013). Especially, IOL rotation of 0° was observed in 7 eyes (23.3%) in Group ZCU and IOL rotation of more than 10° was observed in 3 eyes (10%) in Group 709. There were no significant differences among the 3 groups in ratios of the direction of toric IOL rotation (*P* > 0.05). More clockwise rotation was found in all three groups than counterclockwise rotation.

**Table 3:** Postoperative rotation of toric IOLs.

	Group	Rotation (°)	≤ 5.0°, n(%)	5.0–10.0°, n(%)	> 10°, n(%)
Total	Group ZCU	1.87 ± 1.63	29 (96.7)	1 (3.3)	0 (0)
	Group 709	4.63 ± 4.04	23(76.7)	4 (13.3)	3 (10)
	Group 709 + CTR	4.13 ± 2.10	20 (66.7)	10 (33.3)	0 (0)
Clockwise	Group ZCU	2.36 ± 1.23	14 (46.7)	0 (0)	0 (0)
	Group 709	4.21 ± 3.08	14 (46.7)	4 (13.3)	1 (3.3)
	Group 709 + CTR	4.79 ± 2.04	11 (33.3)	8 (26.7)	0 (0)
Counterclockwise	Group ZCU	2.56 ± 1.67	8 (26.7)	1 (3.3)	0 (0)
	Group 709	5.36 ± 5.41	9 (30)	0 (0)	2 (6.7)
	Group 709 + CTR	3.00 ± 1.73	9 (30)	2 (6.7)	0 (0)

### 3.4 Influencing factors of rotational stability of different toric IOLs

**Fig. 3:**
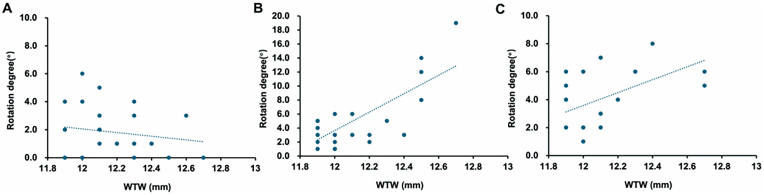
**Relationships between IOL rotation and WTW in the three groups.** Toric IOL rotation is not significantly correlated with WTW in Group ZCU (Pearson *r* = −0.180, *P* = 0.341), whereas positive correlations are present in Group 709 (Pearson *r* = 0.419, *P* = 0.021) and Group 709 + CTR (Pearson *r* = 0.403, *P* = 0.027). Trend lines represent linear regression for each group.

Toric IOL rotation was positively correlated with WTW in Group 709 and Group 709 + CTR (Fig. 3B–C, *r* = 0.419, *P* = 0.021 and *r* = 0.403, *P* = 0.027, respectively), whereas no such correlation was found in Group ZCU (Fig. 3A, *r* = −0.180, *P* = 0.341).

The variables included in the multiple linear regression analysis are age, gender, eye laterality, AL, ACD, LT and WTW. In Group ZCU, no factor was found to be associated with toric IOL rotation. In Group 709, LT and WTW were found to be independently associated with toric IOL rotation (*β* = 0.499, *P* = 0.003 and *β* = 0.390, *P* = 0.018). In Group 709 + CTR, only WTW was found to be independently associated with toric IOL rotation (*β* = 0.418, *P* = 0.002).

## 4 Discussion

Approximately 32.5% of cataract patients have preoperative corneal astigmatism greater than 1 D.^12^ Various surgical techniques, including limbal relaxing incisions, femtosecond laser-assisted arcuate corneal incisions, and toric IOL implantation, have been employed to address this issue.^13^^,^^14^ Among them, toric IOL implantation remains the most effective and durable method, provided that the IOL is accurately aligned.^15^ Even small rotational misalignments can significantly impair astigmatic correction and visual acuity. Each degree of rotation leads to a 3.3% loss in cylindrical power, and rotations exceeding 30° completely negate the cylinder effect, potentially inducing additional astigmatism.^16^^,^^17^

WTW distance, defined as the horizontal corneal diameter, is an important anterior segment anatomical parameter often used to estimate the horizontal dimension of the capsular bag.^18^ Although its direct correlation with capsular bag size remains controversial,^19^^,^^20^ cadaveric studies have suggested a certain degree of association.^21^ Lim et al.^22^ further demonstrated a positive correlation between capsular bag diameter and AL, implying that eyes with long AL may also possess an enlarged capsular bag. This anatomical feature, in turn, may reduce IOL–capsule friction and compromise rotational stability.

This is the first study to compare the rotational stability and astigmatic correction of the ZCU and 709 toric IOLs—with or without CTR implantation—in eyes with WTW >11.8 mm. The ZCU IOL demonstrated significantly superior rotational stability, with a mean rotation of only 1.87° and just one case exceeding 5°, along with significantly lower residual astigmatism and better UCVA, compared to both the 709 and 709 + CTR groups. These findings are in agreement with prior research. For instance, a three-month study reported that the ZCU IOL had significantly lower residual astigmatism (*P* = 0.018), fewer cases of misalignment > 10° (*P* = 0.0044), and reduced prediction error (*P* = 0.043) compared to the TECNIS Toric I (ZCT) IOL, with no need for secondary repositioning.^23^ Chang et al.'s research also observed that all ZCU IOLs remained within 5° of rotation at day 1 and month 3 postoperatively.^24^ However, these studies did not mention the influence of WTW on the included eyes.

Toric IOLs are known to exhibit a certain degree of postoperative rotational misalignment, which remains a key concern for surgeons. A meta-analysis by Li et al.^4^ incorporating various Toric IOL models reported a pooled mean absolute rotation of 2.36° (95% CI: 2.08–2.64), which is higher than the average rotation observed in our study with the ZCU IOL (1.87°). Brar et al.^25^ found that 84% of eyes had ≤ 5° rotation and 16% had 6°–10° rotation at 12 months postoperatively with Eyecryl™ Toric IOLs. Qiu et al.^26^ reported that 5.26% of eyes with SN60T6-T9 IOLs rotated > 10° after one year. Another study on ZCT IOLs found that at 3 months, 68.63% of eyes rotated ≤ 5°, 26.47% rotated 5°–10°, and 5% exceeded 10°.^27^ In contrast, only one eye (3.3%) in our study experienced > 5° rotation, and none exceeded 10°, suggesting relatively better rotational stability of the ZCU IOL in eyes with large WTW.

In contrast to Group ZCU, both Group 709 and Group 709 + CTR showed less favorable rotational stability in eyes with WTW > 11.8 mm, with three cases in Group 709 showing a rotation of more than 10°. Although plate-haptic toric IOLs are traditionally considered more rotationally stable than C-loop designs and are reportedly unaffected by increased AL,^7^^,^^28^^,^^29^ their overall diameter may be inadequate to fully engage the capsular equator in eyes with large WTW, leading to insufficient haptic contact and a higher risk of rotation or decentration. In comparison, the ZCU IOL showed excellent stability even in large-WTW eyes, likely due to its C-loop configuration and frosted haptic design. As reported by Takaku et al.^23^ this modification enhances friction between the haptics and the capsular bag and facilitates faster unfolding, thereby reducing early postoperative rotation and maintaining long-term stability even in relatively loose capsular bags.

Notably, even in Group 709 + CTR, larger WTW distances were associated with greater rotational amplitudes. This is consistent with findings from a recent intraindividual randomized trial by Schartmüller et al.,^30^ which demonstrated that combining a CTR with a plate-haptic toric IOL did not enhance rotational stability. On the contrary, the CTR group exhibited greater decentration, tilt, and axial misalignment compared to the control group.^30^ In our study, the enlarged capsular bag may have further limited the mechanical support provided by the CTR, thereby weakening its ability to counteract the rotational risks caused by preexisting anatomical weaknesses in the capsular structure.

CTRs are commonly used to enhance capsular bag stability by preserving its contour, reducing contraction, and minimizing the potential space between the IOL optic and posterior capsule.^9^^,^^31^ Zhao et al.^32^ reported that toric IOLs showed greater postoperative rotation in eyes with enlarged capsular bags when CTRs were not used. Even with CTR co-implantation, 25% of cases still exhibited 2°–5° of rotation, indicating limited rotational control. To address this, several modified CTR approaches have been explored. Ucar et al.^33^ proposed suturing the CTR to the IOL haptics to increase capsular compression and friction. Jiang et al.^34^ introduced a CTR with additional eyelets to enlarge its contact area with the IOL, enhancing rotational resistance. Some studies also suggest that inserting the CTR after IOL implantation can apply direct pressure to the haptics, improving early postoperative stability.^35^ While these methods show promise in selected high-risk eyes, their efficacy remains influenced by individual ocular anatomy and intraoperative variables. Therefore, in eyes with large WTW or capsular bag expansion, individualized CTR strategies may be necessary to ensure optimal toric IOL stability.

The limitations of this study include its retrospective design, which may introduce surgeon-dependent bias. In addition, toric IOLs with double C-loop or C-plate designs were not included, and patients were not stratified by the timing of CTR implantation (before vs. after IOL insertion). Future prospective randomized studies are warranted to further validate these findings.

## 5 Conclusions

In summary, this study demonstrates that in eyes with WTW > 11.8 mm, the ZCU IOL exhibits superior rotational stability and astigmatic correction compared to the 709 IOL, regardless of CTR use. These findings underscore the need for further optimization of toric IOL designs to provide more personalized surgical strategies for patients with large WTW.

## Study approval

The authors confirm that any aspect of the work covered in this manuscript that involved human patients was conducted with the ethical approval of all relevant bodies, and the study was performed in accordance with the Declaration of Helsinki, and the protocol was approved by Ethics Committee of the Eye and ENT Hospital of Fudan University, Shanghai, China(approval number: 2024015-1).

## Author contributions

The authors confirm contribution to the paper as follows: Concept and design: WH, XZ. Acquisition, analysis, or interpretation of data: All authors. Drafting of the manuscript: SL, WH. Critical revision of the manuscript: WH, XZ. Statistical analysis: SL, WH. Obtained funding: XZ. Supervision: XZ. All authors read and approved the final version of the manuscript

## Funding

This article was supported by the 10.13039/501100012166National Key Research and Development Program of China (2022YFC2502800, 2024YFC2510800), 10.13039/501100001809National Natural Science Foundation of China (82122017, 82271069, 81870642, 82371040, 81970780, 81470613, 81670835 and 82301188), Science and Technology Innovation Action Plan of Shanghai Science and Technology Commission (23Y11909800 and 21S31904900), Outstanding Youth Medical Talents of Shanghai "Rising Stars of Medical Talents" Youth Development Program, 10.13039/501100014137Clinical Research Plan of Shanghai Shenkang Hospital Development Center (SHDC12026129, SHDC12020111), Shanghai Municipal Key Clinical Specialty Program (shslczdzk01901).

## Declaration of competing interest

The authors declare that they have no financial or personal relationships that could inappropriately influence, or be perceived to influence, the research presented in this manuscript.

## Abbreviations

IOL: Intraocular Lens
CTR: Capsular Tension Ring
WTW: White-to-White (corneal diameter)
AL: Axial Length
UCVA: Uncorrected Visual Acuity
BCVA: Best Corrected Visual Acuity
logMAR: Logarithm of the Minimal Angle of Resolution
ACD: Anterior Chamber Depth
LT: Lens Thickness
CCC: Continuous Circular Capsulorhexis
OPD: Optical Path Difference (OPD-Scan III aberrometer)
SD: Standard Deviation
ANOVA: Analysis of Variance
SIA: Surgically Induced Astigmatism
